# Transfer of Tetracycline Resistance Genes with Aggregation Substance in Food-Borne *Enterococcus faecalis*

**DOI:** 10.1007/s00284-014-0742-1

**Published:** 2014-12-07

**Authors:** Jong-Mi Choi, Gun-Jo Woo

**Affiliations:** Laboratory of Food Safety and Evaluation, Department of Food Bioscience and Technology, Korea University, Anam-dong 5-ga, Seongbuk-Gu, Seoul, 136-713 Korea

## Abstract

*Enterococcus faecalis* has the ability to conjugate with the aid of aggregation substance (AS) and inducible sex pheromones to exchange genetic elements in food matrix. To evaluate the food safety condition and the transferable factor, 250 tetracycline-resistant food-borne *E.* *faecalis* were collected in Korea. Among the isolates, a majority of tetracycline-resistant isolates (49.6 %) harbored both the *tet*(M) and *tet*(L) genes together, followed by *tet*(M) (19.6 %), and *tet*(L) (6.8 %) alone. Also, we found the combination of *tet*(L)/*tet*(M)/*tet*(O) or *tet*(M)/*tet*(O). We identified two *tet*(S) genes including the isolate carrying *tet*(M) + *tet*(S) genes. Additionally, most *E. faecalis* were positive for *cpd* and *ccf* (both 96.8 %) followed by *cob* (57.2 %). Through mating experiments, we confirmed *E.* *faecalis* possessing the *Int*-*Tn* gene and/or any AS gene successfully transferred *tet* genes to JH2-2 *E.* *faecalis*, whereas neither *E.* *faecalis* carrying AS genes nor the *Int*-*Tn* gene showed the conjugation. Pulsed-field gel electrophoresis results supported a distinct pattern, implying transfer of genetic information. Our study revealed a high occurrence of tetracycline resistance genes in *E.* *faecalis* from various foods. The widespread dissemination of tetracycline resistance genes would be promoted to transfer tetracycline resistance genes by pheromone-mediated conjugation systems.

## Introduction

Enterococci are found in a diversity of foods as normal inhabitants of the gastrointestinal tracts of food-producing animals and humans [41]. Despite their omnipresence, enterococci have become important nosocomial pathogens and appear to have increasing resistance to antimicrobials [31]. Thus, the prevalence of antimicrobial-resistant enterococci in food-producing animal is becoming a matter of concern, as these resistant bacteria may be transmitted to humans via the food chain [29].

Tetracycline resistance commonly appears as acquired antimicrobial resistance (AMR) in *Enterococcus* [36]. Because tetracycline has been widely used to promote livestock growth and to treat human diseases, the widespread use of this antimicrobial has caused selective pressure and led to an increase in the number of acquired resistance genes among bacteria [30, 38]. Nevertheless, many studies have reported that AMR has persisted due to horizontal transfer of antibiotic resistance [21, 32] and linkage to other classes of antibiotics [23, 34].

*Enterococcus faecalis* possesses unique virulence factors such as aggregation substance (AS) and inducible sex pheromones that can be exchanged as genetic elements by conjugation. Conjugation was first described as a pheromone-responsive conjugation system [14] and allows effective sharing of genetic information such as AMR and virulence factors [9, 17]. Among the pheromone-responsive plasmids, pCF10 plays a significant role in the dissemination of virulence factors and resistance genes among *Enterococcus* spp. [5, 15, 16]. Furthermore, the persistence of enterococci against tetracycline might be related to the pheromone-responsive plasmid pCF10 encoding tetracycline resistance [2]. Plasmid typing method using replication initiation gene (*rep*) sequences have been developed, suggesting the possibility of easy and accurate identification of plasmids [25]. Thus, the purposes of the present study were to investigate the distribution of tetracycline resistance *(tet)* genes in food-borne *E.* *faecalis* in Korea and the factors involved in the pheromone-responsive conjugation system. Additionally, we evaluated the conjugative transferability of *tet* genes associated with the sex pheromone plasmid.

## Materials and Methods

### Bacterial Isolates

250 tetracycline-resistant *E.* *faecalis* isolated from beef (*n* = 47), chicken (*n* = 87), pork (*n* = 65), fish and fishery products (*n* = 47), and processed meat products (*n* = 4) from 2003 to 2010 were provided by National Antimicrobial Resistance Management Program (NARMP) of Korean Food and Drug Administration (Ministry of Food and Drug Safety at present). All the isolates were identified by the VITEK2 Compact (BioMérieuxVitek, Inc., Hazelwood, MO, USA).

### Antimicrobial Susceptibility Test

The antimicrobial susceptibility profiles were determined by disk diffusion and agar dilution methods [10]. Nine antibiotics were used as follows: ampicillin (10 µg), vancomycin (30 µg), teicoplanin (30 µg), erythromycin (15 µg), ciprofloxacin (5 µg), chloramphenicol (30 µg), linezolid (30 µg), gentamicin (120 µg), and streptomycin (300 µg) (BD Sensi-disc, Becton–Dickinson, Mannheim, Germany). Tetracycline was diluted to minimum inhibitory concentrations (MICs) of 0.06–256 μg/ml to determine the degree of the tetracycline resistance in the isolates, and the data were interpreted according to CLSI guidelines [10]. *Staphylococcus aureus* ATCC 25923, *E.* *faecalis* ATCC 29212, and *E.* *faecalis* ATCC 51299 were used as control strains for the disk diffusion and MIC tests, respectively.

### Polymerase Chain Reaction

Of the 250 tetracycline-resistant isolates, polymerase chain reaction (PCR) was carried out to determine the presence of genes encoding tetracycline resistance [*tet*(K), *tet*(L), *tet*(M), *tet*(O), *tet*(S), *tet*(T), and *tet*(W)] and the Tn*916*–*1545* transposon family for the integrase (*Int*-*Tn*) gene. AS genes [*agg, asa1, prgB,* *and asa373*], inducible pheromones [*cpd*, *cob*, and *ccf*], and the *rep* gene [*prgW*] were also detected. The primers and PCR conditions used are shown in Table 1.

**Table 1:** Primers and PCR conditions used in the present study

Description	Target gene	Primer sequence (5′ → 3′)	Product Size (bp)	Reference
Tetracycline resistance	*tetK*	F: TTAGGTGAAGGGTTAGGTCC	718	[1]
		R: GCAAACTCATTCCAGAAGCA		
	*tetL*	F: ATAAATTGTTTCGGGTCGGTAAT	1,077	[40]
		R: AACCAGCCAACTAATGACAATGAT		
	*tetM*	F: GTTAAATAGTGTTCTTGGAG	657	[1]
		R: CTAAGATATGGCTCTAACAA		
	*tetO*	F: GATGGCATACAGGCACAGAC	614	
		R: CAATATCACCAGAGCAGGCT		
	*tetS*	F: TGGAACGCCAGAGAGGTATT	660	
		R: ACATAGACAAGCCGTTGACC		
	*tetT*	F: AAGGTTTATTATATAAAAGTG	169	[3]
		R: AGGTGTATCTATGATATTTAC		
	*tetW*	F: GAGAGCCTGCTATATGCCAGC	168	
		R: GGGCGTATCCACAATGTTAAC		
Aggregation substance	*agg*	F: AAGAAAAAGAAGTAGACCAAC	1,553	[18]
		R: AAACGGCAAGACAAGTAAATA		
	*asa1*	F: CACGCTATTACGAACTATGA	375	[6]
		R: TAAGAAAGAACATCACCACGA		
	*prgB*	F: ATACAAAGCCAATGTCG	427	[20]
		R: TACAAACGGCAAGACAAG		
	*asa373*	F: GGACGCACGTACACAAAGCTAC	619	[11]
		R: CTGGGTGTGATTCCGCTGTTA		
Tn*916*–*1545* family integrase	*Int*-*Tn*	F: GCGTGATTGTATCTCACT	1,028	[12]
		R: GACGCTCCTGTTGCTTCT		
Sex pheromone	*cpd*	F: TGGTGGGTTATTTTTCAATTC	782	[18]
		R: TACGGCTCTGGCTTACTA		
	*cob*	F: AACATTCAGCAAACAAAGC	1,405	
		R: TTGTCATAAAGAGTGGTCAT		
	*ccf*	F: GGGAATTGAGTAGTGAAGAAG	543	
		R: AGCCGCTAAAATCGGTAAAAT		
*Rep* ^a^ of pCF10	*prgW*	F: GCTCGATCARTTTTCAGAAG	201	[25]
		R: CGCAAACATTTGTCWATTTCTT		

^a^Replication initiator protein gene

Amplification reactions were performed in a total volume of 30 µl containing 15 µl of PCR pre-mix with *Taq* DNA polymerase (Solgent, Seoul, Korea), 2 µl of bacterial template DNA, 1 µl of 10 pmol of each primer, and 11 µl of ultrapure distilled water. The PCR products were visualized on 1.5 % agarose gels (Promega, Madison, WI, USA) stained with ethidium bromide using the Gel Doc system (Bio-Rad, Hercules, CA, USA). Positive controls were used with *E.* *faecalis* ATCC 29212 (*cpd*, *cob*, and *ccf*) for each target gene, or the sequences were analyzed by Macrogen Inc. (Seoul, South Korea). The sequences were analyzed using the GenBank database of the National Center for Biotechnology Information and the BLAST search engine (http://www.ncbi.nlm.nih.gov/BLAST).

### Transferability Test by Filter Mating

The selected tetracycline-resistant isolates were tested for transferability by filter mating [8]. *E.* *faecalis* JH2-2 was used as the plasmid-free recipient strain [24]. Filter mating was conducted using a 1:10 donor–recipient mixture. Five ml of overnight culture was mixed and harvested for 4 h. The mixture was poured on a 0.45-µm filter membrane and incubated on brain heart infusion (BHI) agar plates at 37 °C overnight. The membrane was diluted in sterile saline (0.85 % NaCl) and spread on a selective BHI agar plate with 10 µg/ml tetracycline, 50 µg/ml rifampin, and 100 µg/ml fusidic acid (triple selective medium). The agar plates were incubated for 24 h at 37 °C, and the typical transconjugants were selected.

### Pulsed-Field Gel Electrophoresis (PFGE)

The genetic relationships among tetracycline-resistant *E.* *faecalis* harboring at least one or more *tet* genes were evaluated based on PFGE carried out with the CHEF-Mapper system (Bio-Rad) [37]. Genomic DNA was digested with 20 U *Sma*I (Takara Bio, Kyoto, Japan) and separated on 1.0 % pulsed-field certified agarose (Bio-Rad). Running conditions were 6.0 V/cm at 14 °C for 20 h with pulse times ramped from 1 to 20 s in 0.5 × TBE buffer. A lambda DNA ladder (Bio-Rad) was used as the size marker. A cluster analysis of the PFGE results was conducted to determine relatedness of tetracycline-resistant isolates using the InfoQuest FP Software version 4.5 (Bio-Rad) with the Dice co-efficient and the unweighted pair group method with arithmetic averages. Optimization settings for the dendrogram were 0.5 % with a band tolerance of 0.1 %.

## Results

### Antimicrobial Resistant Profiles

Tetracycline-resistant *E.* *faecalis* isolates were resistant to ciprofloxacin (29.2 %), streptomycin (29.2 %), erythromycin (27.6 %), chloramphenicol (18.0 %), and linezolid (16.4 %). 8.8 % of the isolates were resistant to gentamicin. None of the identified isolates was resistant to ampicillin, vancomycin, and teicoplanin. High level of tetracycline resistance (128–256 µl/ml) was observed in *E.* *faecalis* isolates from chicken, pork, fish and fishery products (Table 2).

**Table 2:** Characterization of MIC, tetracycline resistance genes and virulence traits in food-borne *Enterococcus faecalis*

Origin (No. of isolates)	TE MIC (µg/ml)	No. of isolates (%)												
		TE resistance genes^a^							*Int*-*Tn* ^c^	Virulence traits				*Rep* ^e^ of pCF10 plasmid
		*Tet*(L)	*tet*(L) +*tet*(M)	*Tet*(M)	*Tet*(S)	*Tet*(L) +*tet*(M) +*tet*(O)	*tet*(M) +*tet*(O)	*tet*(M) +*tet*(S)		AS^d^	Inducible sex pheromones			
										*agg*	*cob*	*ccf*	*cpd*	*prgw*
Beef (47)	32–128	4	20	18	ND^b^	ND	ND	1	16	18	25	46	43	36
Chicken (87)	32–258	3	40	33	ND	7	1	ND	48	49	41	86	87	83
Pork (65)	16–256	6	39	15	1	1	ND	ND	23	18	29	62	63	49
Fish and fishery products (47)	32–256	2	25	12	ND	ND	ND	ND	19	24	47	46	47	42
Meat processed products (4)	64–128	2	ND	1	ND	ND	ND	ND	1	0	1	2	2	1
Total (250)		17 (6.8)	124 (49.6)	79 (31.6)	1 (0.4)	8 (3.2)	1 (0.4)	1 (0.4)	10 (42.8)	10 (43.6)	14 (57.2)	24 (96.8)	242 (96.8)	211 (84.4)

^a^Tetracycline

^b^Not detected

^c^Tn *916*–*1545* family integrase gene

^d^Aggregation substance

^e^Replication initiation gene

### Distribution of *tet* Genes

All except 19 isolates in this study carried at least one of the *tet* genes. Table 2 shows the tetracycline-resistant gene patterns. In total, 124 tetracycline-resistant isolates carried both the *tet*(M) and *tet*(L) genes together. Only 17 isolates had *tet*(L) gene alone, and the remaining 79 isolates were positive only for *tet*(M) gene. The combination of *tet*(L), *tet*(M), and *tet*(O) appeared in eight *E.* *faecalis* isolates, whereas one isolate was detected with both *tet*(M) and *tet*(O). We found two *tet*(S) genes that have been rarely found in food-borne *E.* *faecalis* isolates including *tet*(M) + *tet*(S). The *tet*(M) gene was the most frequently found among *E.* *faecalis*, followed by the *tet*(L) gene. 107 isolates (46 %) possessed the *Int*-*Tn* gene carrying the transposon of the Tn*916*–*1545* family. Of them, 104 isolates (97 %) of the *tet*(M)-carrying isolate were positive for Tn*916*–*1545* element (data not shown here).

### Distribution of Virulence Genes

The *agg* gene was detected in 109 (43.6 %) of 250 tetracycline-resistant isolates through the use of the highly conserved sequence of the pheromone-responsive plasmids (pAD1, pPD1, and pCF10) [18]. *E.* *faecalis* isolates were positive for *cpd* (96.8 %), *ccf* (96.8 %), and *cob* (57.2 %), respectively. At least one or more inducible sex pheromones were detected in all the isolates, and the *agg* virulence determinant was present with all pheromone determinants. *prgW* (84.4 %) gene was identified in 84.4 % of isolates (Table 3).

**Table 3:** Characterization of transconjugants derived from tetracycline-resistant food-borne *Enterococcus faecalis*

Isolates	Origin	Donor strains			*E.* *faecalis* JH2-2		
		AS^a^	TE^b^ resistance genes	*Int*-*Tn* ^c^	AS	TE resistance genes	*Int*-*Tn*
EFS25-FB-KF03	Beef	*prgB, asa1, asa373*	*tet*(L)	–	*prgB, asa1, asa373*	*tet*(L)	–
EFS65-FB-KF04	Beef	NT^d^	*tet*(L)	–	NT		
EFS30-FB-KF03	Beef	*prgB, asa1*	*tet*(M)	+	*prgB, asa1*	*tet*(M)	+
EFS333-FP-KF09	Pork	NT	*tet*(M)	–	NT		
EFS45-FC-KF04	Chicken	*asa1*	*tet*(M) + *tet*(O)	+	*asa1*	*tet*(M) + *tet*(O)	+
EFS37-FC-KF03	Chicken	*prgB, asa1*	*tet*(L) + *tet*(M)	–	*prgB, asa1*	*tet*(L) + *tet*(M)	–
EFS365-FP-KF10	Pork	NT	*tet*(L) + *tet*(M)	+	NT	*tet*(L) + *tet*(M)	+
EFS113-FP-KF04	Pork	*asa1*	*tet*(L) + *tet*(M) + *tet*(O)	+	*asa1*	*tet*(L) + *tet*(M) + *tet*(O)	+
EFS399-FC-FK10	Chicken	*asa1*	*tet*(L) + *tet*(M) + *tet*(O)	–	*asa1*	*tet*(L) + *tet*(M)	–
EFS405-FP-KF10	Pork	*prgB, asa1*	*tet*(S)	–	*prgB, asa1*	*tet*(S)	–
EFS438-FB-KF10	Beef	*prgB, asa1*	*tet*(M) + *tet*(S)	–	*prgB, asa1*	*tet*(M) + *tet*(S)	–

^a^Aggregation substance protein genes

^b^Tetracycline

^c^Tn*916*–*1545* family integrase gene

^d^Not transferred

### Identification of Gene Transferability

The transferability was identified in 11 selected isolates (Table 3), and confirmed to transfer to JH2-2 *E.* *faecalis* from 5(83.3 %) strains of *tet*(L), 7(87.5 %) strains of *tet*(M), 2(66.7 %) strains of *tet*(O), and 2(100 %) strains of *tet*(S). Of the AS genes, most isolates carried *asa1* (72.7 %), *prgB* (45.4 %), and *asa373* (9.1 %). All AS genes of donor strains were simultaneously and completely transferred to JH 2-2 *E.* *faecalis*. The *Int*-*Tn* (36.4 %) gene was found in four isolates, and all *Int*-*Tn* genes were transferred to JH 2-2 *E.* *faecalis* with *tet* genes.

### Analysis of Genetic Relationship by PFGE

**Fig. 1:**
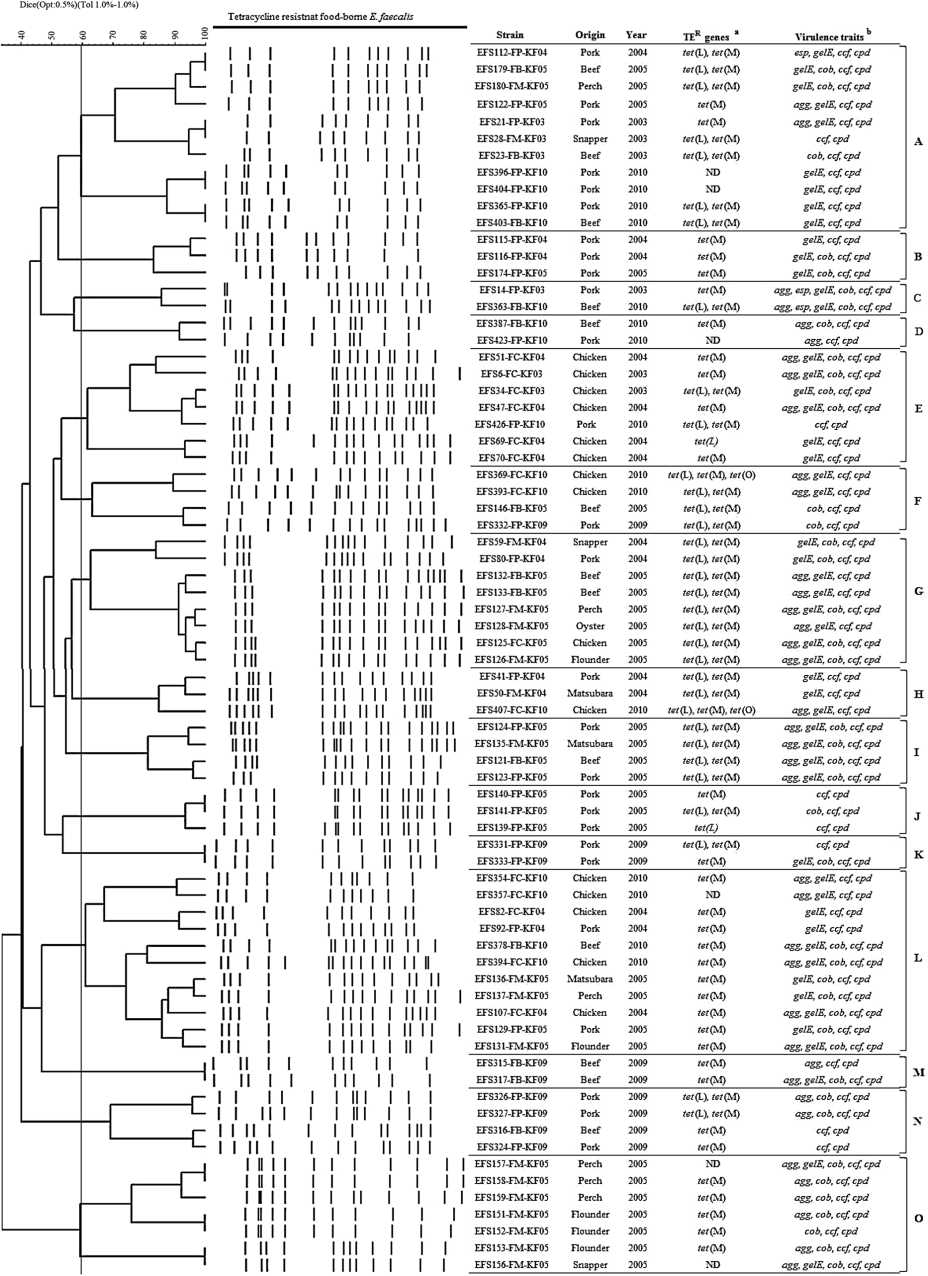
Pulsed-field gel electrophoresis (PFGE) dendrogram of tetracycline-resistant *Enterococcus faecalis* originating from retail beef, chicken, pork, fish and fishery products, and processed meat products *a* tetracycline resistance genes *b* *agg*, *gelE*, and *esp* were detected in a previous study (unpublished data) *c* not detected

The genetic relationships among the 250 tetracycline-resistant *E.* *faecalis* isolates were evaluated based on PFGE with *Sma*I restriction digestion. In Fig. 1, clusters consisted of 73 isolates showing the related PFGE types based on 80 % similarity cut-off in the 250 *E.* *faecalis* isolates. Isolates with over 80 % similarity were clustered again at 60 % similarity cut-off. 15 groups were assigned as new clusters (A–O), with the same or similar patterns of *tet* genes and virulence factors. Cluster A and cluster L showed high genetic homology (over 90 %) compared to other clusters. Especially, the sub-grouping was confirmed by the year of isolation 2003, 2004–2005, and 2010. Clusters B, J, K, and N represented clones that contained isolates from mainly pork origins. The isolates in cluster G and I only harbored *tet*(L) and *tet*(M). Isolates in cluster I showed 100 % similarity in virulence profiles. Fishery product isolates generated cluster O, carrying *tet*(M) except for two isolates without any *tet* gene.

## Discussion

One of the main concerns regarding enterococci is their potential role as a reservoir for AMR and virulence traits that can be disseminated to other bacteria. Many of these factors have been found in enterococci, as shown in this study.

Tetracyclines have been used for various purposes; however, overuse of antibiotic causes selective pressure and has helped that bacteria acquire resistance genes [30, 38]. The mechanism of tetracycline resistance has been described as the effect of efflux pumps [*tet*(K), *tet*(L)] and ribosomal protection proteins [*tet*(M), *tet*(O), *tet*(S), *tet*(T), and *tet*(W)] [35]. In a previous study, resistance mediated by *tet*(M) was reported to be the most frequent in the isolates from food animals [1], whereas *tet*(L) is the most frequent determinant responsible for tetracycline resistance enterococci from food [22]. Food-borne *E.* *faecalis* containing *tet*(M) and *tet*(L), with an MIC range of 128–256 µl/ml against tetracycline resistance, were frequently detected in this study. The expression of high-level tetracycline resistance might help to explain the complementary mechanism of the efflux pump and ribosomal proteins [4].

Besides, we investigated the enterococcal virulence traits including adherence to tissue, invasion formation, and inducible pheromones. Several genes representing the traits have been characterized as enhancer to exchange genetic information such as transmissible antibiotic resistance plasmids or virulence factors by conjugation. The process has been also known to take place in gastrointestinal tract [17]. Among the virulence genes, we found *agg* gene in 109 tetracycline-resistant isolates. The prevalence of *agg* supports the transfer of tetracycline resistance to an inducible pheromone plasmid. A*gg*, targeting the highly conserved region of the sex pheromone plasmids pAD1, pPD1, and pCF10, is a unique virulence trait in *E.* *faecalis* that allows horizontal transfer of antibiotic resistance and virulence genes at high frequency. In this study, *agg* positive isolates possessed AMR and virulence traits respectively (data not shown here). To demonstrate transfer/acquisition ability of the isolates harboring AS genes, we selected the isolates carrying at least one or more tetracycline resistance genes, then conducted filter mating using *E.* *faecalis* plasmid-free strain JH2-2. The results showed that the isolates possessing the transposon elements successfully generated transconjugants by transferring tetracycline resistance genes. They could also transfer their *tet* genes, possessing AS genes without transposon elements, which might become activated with the conjugative sex pheromone system and ASs. Of the AS genes, *asa1* was the most commonly found, followed by *prgB* gene. The *asa1* gene encoding AS of the pheromone-responsive plasmid pAD1 has been well characterized, and the presence of AS genes in *Enterococcus* results in fast conjugation [43]. The *prgB* gene, encoding the surface protein, mediates cell aggregation by conjugative transfer of the pheromone-responsive plasmid pCF10 in *E.* *faecalis* which promotes conjugation to share pathogenic information [7, 13]. In our study, the transconjugant isolates had at least one AS gene transferred simultaneously by conjugation with the *tet* genes. Interestingly, two *tet*(S) genes were transferred to *E.* *faecalis* JH2-2, although they were not associated with effective vehicles such as Tn*916*–*1545* for *tet*(M) and *tet*(S). *tet*(S) is transferred from chromosome to chromosome of other *E.* *faecalis* isolates by conjugation [19]. It suggests that the transfer mechanism on the chromosome is based on movement of the pAD1 and pCF10 plasmids [28]. Therefore, successful conjugation may have been caused by the AS genes (*asa1* and *prgB*) in the two isolates and activated to involve pheromone-inducible plasmids for conjugation. In addition, detection of *tet*(S) in *E.* *faecalis* from pork in this study is the first report in Korea. In general, *tet*(S) gene is not detected in food-producing animal. *tet*(S) in *Vibrio* sp. from fish was reported in Korea [26]. Besides, the presence of the AS gene *asa373* is important in Enterococci. A low incidence of *asa373* in Enterococci were found and suggested a correlation between *asa1*, *asa373,* and *esp* [42]. We found a positive link among *asa1*, *prgB*, and *asa373* in donor and transconjugant isolates. Our PFGE results supported the transfer of these genes. The PFGE analysis pattern shows transfers by genetic mobile elements such as tetracycline resistance genes, conjugative transposons, and pheromone-inducible plasmids. In addition, several clones showed complete consensus in pheno/genotype profiles in different food sources. Among the isolates showing clonality, food-borne *E.* *faecalis* isolated in 2005 were highly prevalent, suggesting that serious cross-contamination had occurred during the process of transport or sale steps. For instance, the isolates belonging to cluster I were analyzed with a high consensus of genetic information despite of different origins.

The *cpd*, *cob*, and *ccf* inducible sex pheromone determinants were found in more than one *E.* *faecalis* isolate tested. These pheromones are relevant to the problems associated with AS genes [17]. The isolates harboring *agg* respond to the recipient *E.* *faecalis* by producing the pheromones to acquire pheromone-inducible plasmids, indicating that inducible sex pheromone-producing *E.* *faecalis* can increase virulence traits as well as antimicrobial resistance by acquiring the plasmid [18]. In the present study, all food-borne *E.* *faecalis* had the ability to acquire sex pheromone plasmids. Among the detected pheromones, the *ccf* gene activates the conjugation of the pCF10 plasmid. The pheromone responsive pCF10 plasmid has been associated with the dissemination of tetracycline resistance among *Enterococcus* [2]. Additionally, several studies have shown that pheromone-mediated conjugation systems are associated with acquiring glycopeptide resistance by mating experiments. Through the mating experiments, several studies have revealed that vanA conjugative plasmid is associated with pheromone-responsive pCF10 [20, 27, 33, 39]. Therefore, the high prevalence of pCF10 in food-borne *E.* *faecalis* indicates that various foods might be potential pathogenic factors to acquire multi-antimicrobial resistance genes and virulence traits and act as an effective vehicle for spreading the pathogens.

We must take into account a limitation of this study, the low number of tetracycline-resistant genes. Due to this limitation, it was difficult to draw conclusion regarding the genetic relationship among tetracycline-resistant genes in the isolates. This issue requires further investigation.

The use of antibiotics as feed additives was partially banned in 2005, and tetracyclines were completely banned for use as feed additive in livestock to reduce antibiotic resistance in 2009 in Korea. However, our results show a high occurrence of tetracycline resistance genes, and the diversity of food sources (retail meat, fish, fishery products, and processed meat products) are still functioning as huge reservoirs for tetracycline resistance and virulence factors as well. We showed that the wide dissemination of pathogenic traits might be promoted by transfer of pheromone-mediated conjugation systems. Therefore, a continuous monitoring is needed at the national level such as NARMP in Korea to check the effect of antibiotics as feed additives to decrease antimicrobial resistance and determinants in food-borne pathogens after complete banning of antibiotics as feed additives in Korea.
